# Combining Molecular Dynamic Information and an Aspherical-Atom Data Bank in the Evaluation of the Electrostatic Interaction Energy in Multimeric Protein-Ligand Complex: A Case Study for HIV-1 Protease

**DOI:** 10.3390/molecules26133872

**Published:** 2021-06-24

**Authors:** Prashant Kumar, Paulina Maria Dominiak

**Affiliations:** Biological and Chemical Research Centre, Department of Chemistry, University of Warsaw, Ul. Żwirki i Wigury 101, 02-089 Warszawa, Poland; pkumar@chem.uw.edu.pl

**Keywords:** electrostatic interactions, protein–ligand interactions, quantum crystallography

## Abstract

Computational analysis of protein–ligand interactions is of crucial importance for drug discovery. Assessment of ligand binding energy allows us to have a glimpse of the potential of a small organic molecule to be a ligand to the binding site of a protein target. Available scoring functions, such as in docking programs, all rely on equations that sum each type of protein–ligand interactions in order to predict the binding affinity. Most of the scoring functions consider electrostatic interactions involving the protein and the ligand. Electrostatic interactions constitute one of the most important part of total interactions between macromolecules. Unlike dispersion forces, they are highly directional and therefore dominate the nature of molecular packing in crystals and in biological complexes and contribute significantly to differences in inhibition strength among related enzyme inhibitors. In this study, complexes of HIV-1 protease with inhibitor molecules (JE-2147 and darunavir) were analyzed by using charge densities from the transferable aspherical-atom University at Buffalo Databank (UBDB). Moreover, we analyzed the electrostatic interaction energy for an ensemble of structures, using molecular dynamic simulations to highlight the main features of electrostatic interactions important for binding affinity.

## 1. Background

The aim of structure-based drug design is to predict which potential drug molecules will bind with high affinity and specificity to the target molecule, using structural data. Generally, the target molecule is a protein or nucleic acid of known or predicted structure. The target molecule can be a mutated or abnormally produced wild human molecule, an essential component of an infectious agent, such as a virus, or a toxin. Ideally, the potential drug will be a small soluble compound. Several new drugs have been developed, with designs guided by the crystal structures of proteins, especially for compounds that inhibits the action of enzymes.

HIV protease is a well-validated target for AIDS control. Crystal structures of HIV protease with different inhibitors and molecular models were used to guide the design of antiviral drugs for AIDS. However, the present challenge is to overcome the rapid development of drug-resistant strains of HIV [1]. Mutations have been observed in more than half of the protease residues on exposure to drugs, and, in general, multiple mutations are found to have high level of resistance [2]. Consequently, the newer drugs must be designed to target different combinations of possible protease mutants.

HIV protease is an aspartate protease existing as a homodimer, and each monomer contains 99 amino acid residues. The homodimer is the functional state of the enzyme. The active site lies between the identical subunits and has the characteristic Asp-Thr-Gly (Asp25, Thr26, and Gly27) sequence. The Asp-(Thr/Ser)-Gly sequence is conserved in the active sites of entire aspartic proteases family, including some other retroviral proteases and pepsin-like proteases, e.g., human T-cell leukemia virus type 1 (HTLV-1) protease, [3] human pepsin [4], and memapsin 2 [5]. The two Asp25 residues (one from each chain) act as the catalytic residues. According to the mechanism for HIV PR protein cleavage, water acts as a nucleophile, which acts in simultaneous conjunction with a well-placed aspartic acid to hydrolyze the scissile peptide bond [6].

Currently nine HIV-protease inhibitors have been approved by the FDA (indinavir, saquinavir, nelfinavir, ritonavir, amprenavir, lopinavir, atazanavir, tipranavir, and darunavir). All of these inhibitors can lose their activity when confronted with mutants. Understanding the cause for loss of potency can give new insight for drug designers to develop more improved inhibitors of HIV-protease. There are at least 50 mutation positions identified within the HIV-1 protease [7,8,9,10,11]. From a structural point of view, these mutations have been classified as active site and non-active site, depending on whether they are located within or outside the active site cavity [12]. In general, most major mutations occur within the active site and are very conservative; that is, they preserve the charge and polarity and only alter the geometry of the active site. These constraints are dictated by the requirement that the enzyme needs to maintain a sufficient affinity for the substrate and a viable catalytic activity. Because non-active site mutations do not directly affect inhibitor/protease interactions, their effects need to be traced back to a chain of events throughout the protease structure. Originally their role was assumed to be only of a compensatory nature, but recent evidence indicates that some of them might play a very important role in lowering the affinity of inhibitors [13,14].

Here we would like to focus on electrostatic component of intermolecular interactions between HIV-1 protease and inhibitors. To compute energy of this component, we selected a method which is based on the assumption that atomic electron densities, expressed as a superposition of spherical harmonic functions, are transferable between atoms in chemically identical environments. Thus, a databank of transferable aspherical atomic densities can be built and used to reconstruct (macro)molecular electron densities in fast but accurate fashion. Such an approach eliminates many of the approximations that are inherent in the point-charge (PC) model commonly used in classical force fields. The point charge model has several limitations. It is unable to take into account such phenomena as electron polarization, subtle details of electron density anisotropy, and charge penetration. Considerable efforts have been devoted to provide a more realistic description of intermolecular interactions by including the aforementioned aspects with recent emphasis on penetration energy (Epen) [15,16,17,18,19,20,21,22,23,24,25]. Here we based our analysis on the University at Buffalo Databank (UBDB) of transferable atomic densities [26,27,28], from which the charge densities of protease–inhibitor complexes was reconstructed. Electrostatic interaction energies were then evaluated by using an exact integration algorithm for the short-range interactions and the Buckingham approximation for non-overlapping densities for atoms at large distances [29].

Electrostatic interaction energies are usually the largest components of the interaction energy, followed by energies of exchange-repulsion, induction, and dispersion. Even if electrostatic interactions are not always the strongest one, due to being long-range and often directional, they make a dominant contribution to the relative orientation of the enzyme and substrate and therefore to molecular recognition and substrate specificity [30,31,32,33,34]. Electrostatic energies have also been shown to be a more reliable indicator of relative biding strength than more accurate total energies when dealing with questionable geometries [35]. The remaining components of total energy are by far sensitive to errors in atomic positions when dealing with interactions close to equilibrium geometry. Thus, electrostatics may serve as a scoring function when working with crystallographic structures or structures resulting from molecular dynamics.

**Figure 1:**
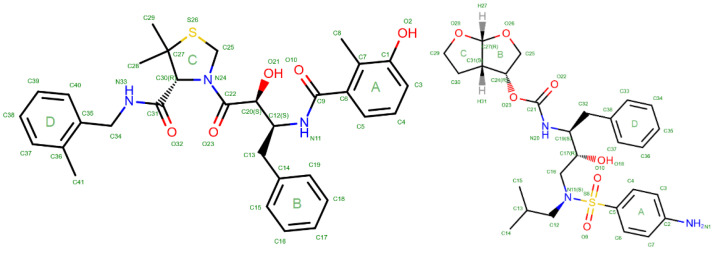
Two-dimensional diagram along with the numbering scheme of JE-2147 (**left**) and darunavir (**right**) molecules according to the RCSB PDB service.

In this research paper, we report a molecular dynamics (MD) simulation study followed by of a comprehensive electrostatic interactions evaluation for binding of inhibitors (JE-2147 and darunavir, Figure 1) to the wild-type HIV 1 proteases and simultaneously for monomer–monomer interactions in these homodimeric proteins. In addition, we have traced the crucial role of penetration energy in ligand and monomer–monomer binding. We included dynamic component of the complex by computing interaction energies for 100 snapshots from MD and analysing averaged values of energies and their deviations from the average. As a result, we provide deeper understanding of electrostatic interactions in HIV-1 protein complexes and propose that introducing the electrostatic component analysis in drug design may allow us to construct protocols to find drug molecules effective against the HIV protease resistant variants, too.

## 2. Theory and Computation Details

### 2.1. Molecular Dynamics (MD) Simulation

Molecular dynamics (MD) simulations were performed to simulate the dynamic behavior of protein in order to reconstruct snapshots of simulated protein electron density and compute pairwise electrostatic energy calculation at the atom level which can be further aggregated at residue and chain level. For MD simulation, the crystal structures of the HIV-1 protein (1KZK 1.09 Å and 4DQB 1.5 Å) [36,37] were obtained from the RCSB database. H++ server was used to add hydrogen atoms according to the specified pH of the environment for the protein structure [38]. The HF/6-31G* ab initio level calculations were performed to optimize ligand geometry (JE-2147 and darunavir, respectively) and obtain the electrostatic potential of the ligand, using the GAUSSIAN16 program [39]. To derive the equivalent partial atomic charges for the ligand, RESP [40] fitting was applied on the electrostatic potentials using the ANTECHAMBER program [41]. To assign the atom types, bond, angle, dihedral, and van der Waals parameters for the ligand, general AMBER force field (GAFF) [42] was used within the ANTECHAMBER program. The LEaP module of Amber18 suite [43] was used to prepare the complex for simulation. FF14SB [44], containing all atom force fields, was used to parametrize protein atoms. ASP25B was kept protonated [45,46,47,48]. Both complexes were neutralized with Cl ions (7 and 8 for the 1KZK and 4DQB structures, respectively), followed by solvating 10 Å buffer of TIP3P [49] water molecules around the system in each direction, forming a truncated octahedral shaped ice cube.

The solvent was first minimized for 1000 steps followed by full system minimization with 4000 steps. Then the system was heated to 298.15 K over 60 ps with a 2 fs step running a further 100 ps MD run for equilibrium. Once an initial equilibrium was reached, with the temperature and density both being stable, the final stage of the simulation was performed. This consists of running a production simulation at 298.15 K for 2 fs. The system was then run for 10 ns, and stability of trajectory was closely monitored. During simulation, the long-range electrostatic interactions were treated by using the partial-mesh Ewald method [50]. For short-range non-bonded interactions, a 10 Å cutoff was employed. The temperature and pressure were kept constant in the simulation by coupling to the system with Berendsen’s thermostat and barostat, respectively [51]. Bond lengths between hydrogens and heavy atoms were constrained by using SHAKE [52]. All production-phase simulations were run by using GPU (NVIDIA Tesla K40 XL) accelerated particle-mesh Ewald molecular dynamics (PMEMD), as implemented in Amber18. For the analysis, 100 snapshots were extracted from the 10 ns MD trajectory for the system (after stripping all waters and ions).

### 2.2. Electrostatic Interaction Energy

To compute the pairwise electrostatic interaction energy (Ees), and penetration contribution to it (Epen), using the UBDB approach, the molecular electron density was represented by Hansen atom model (Equation (1)) and parameters of the model specific for each atom type were reproduced from the UBDB2018 [28]. The LSDB program was used to transfer the multipole populations from the UBDB to all snapshot structures. This transfer was based on the atomic connectivity and local symmetry recognition. The XDPROP module of the XD2016 package [53] was used to calculate interaction energies from the derived charge density, using the Exact Potential Multipole Method (EPMM) [54]. The EPMM evaluates the exact Coulomb integral in the inner region (≤4.5 Å) and combines it with a Buckingham-type multipole approximation for long-range interatomic interactions.

(1) $$\rho_{k}\left(r\right)=\underset{\rho_{core}}{\underbrace{P_{c}\rho_{c}\left(r_{k}\right)}}+\underset{\rho_{valence}}{\underbrace{P_{v}\kappa^{3}\rho_{v}\left(\kappa r_{k}\right)}}+\underset{\rho_{deformation}}{\underbrace{\overset{l_{max}}{\underset{l=0}{\sum }}\kappa^{\prime 3}R_{l}\left(\kappa \prime r_{k}\right)\overset{l}{\underset{m=0}{\sum }}P_{lm\pm }y_{lm\pm }\left(\theta ,\varphi \right)}}$$

To compute the pairwise electrostatic interaction energy (Ees) by using the point-charge (PC) model, the same atomic point charges were used which were applied in molecular dynamic simulations. The charges were transferred from the Amber18 suite files to the XD2016 package files by the homemade scripts. The interaction energy was calculated by the XDPROP module of the XD2016 package.

## 3. Results and Discussion

### 3.1. HIV-1 Protease Interactions with Ligand

The trajectory obtained from MD simulation show stable protease complex with 1.815 Å and 1.49 Å average RMS deviations for 1KZK and 4DQB structures, respectively. The low RSMD values suggest good stability of the complexes.

**Figure 2:**
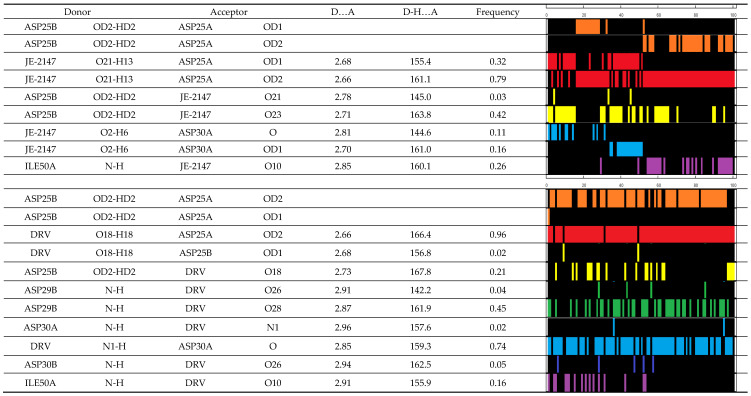
Hydrogen bonds identified in 100 snapshots of MD simulations for 1KZK (**top**) and 4DQB (**bottom**) structures, including average values of donor–acceptor distances (A) and hydrogen bond angles (°), along with the graphical representation of their occurrence (colored bars) in particular snapshot.

From the MD analysis, it was found that two hydrogen bonds that were stable over the entire simulation were formed between JE-2147 ligand and protein (Figure 2, top). These are bonds with Asp25A (O21-H13…OD2) and Asp25B (OD2-HD2…O23) residues. There are also hydrogen bonds which alternate with each other. Bonds with Asp25A OD1 and Asp30A O or Asp30A OD1 exist only in the first half of the simulation, and in the remaining half bonds with Ile50A N-H are formed. This is related to alternations of hydrogen bonding between two catalytic residues: Asp25B OD2-HD2 interacts either with OD1 (first half of simulation) or OD2 of Asp25A.

Hydrogen bonds and other contacts contributed to total energy of ligand interaction with the protein. The average value of electrostatic interaction energy, computed on the basis of 100 snapshots from MD simulations, amounts to −328(68) kJ/mol for interactions with entire protein, and to −210(42) and −118(46) kJ/mol for interactions with chain A and B, respectively.

**Figure 3:**
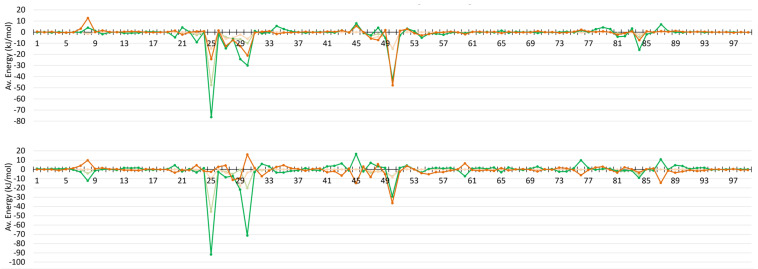
Averaged electrostatic interaction energies (Ees, kJ/mol) and penetration contributions to them (Epen, kJ/mol) for interactions of a ligand with each residue of a monomer for 1KZK (**top**) and 4DQB (**bottom**) structures. Ees: dark green—monomer A; dark orange—monomer B. Epen: light green—monomer A; light orange—monomer B.

When electrostatic contributions of each residue to the binding with the ligand are analyzed (Figure 3) it become visible that residues forming above identified hydrogen bonds contribute the most. The largest contribution comes from Asp25A (−76(23) kJ/mol), which could also be envisioned from proton donor and acceptors distances, the shortest ones among observed. The next largest contributions result from interactions with Ile50A (−43(17) kJ/mol) and surpassingly with Ile50B (−48(14) kJ/mol). The later residue was not identified during geometric analysis to form important hydrogen bonds. Manual inspection of structures showed that Ile50B N-H interacts with JE-2147 O32, although with slightly longer that commonly accepted donor–acceptor distances. Moreover, judging from donor–acceptor distance, interactions with the Ile50 residues would not be the next strongest identified. Apparently, energetic criterion helps better to understand which residues are important for binding than geometric and only focused on hydrogen bonding criteria. A similar situation appears for next strong electrostatic attractions, i.e., interactions with Asp30A (−30(30) kJ/mol) and surpassingly also with Asp30B (−21(7) kJ/mol). The later forms weaker interactions, but energetically more stable over the simulations and is capable to form C-H–O interactions with the O oxygen from Asp30B and the C37-H, C38-H, or C41-H groups from the D phenyl ring of ligand, or N-H–pi interactions with that same phenyl ring. Following this path, the next important contribution identified comes from Asp29A and Asp29B, whose residues tend to form N-H–pi interactions with the aromatic rings of the ligand (the A phenyl ring: −24(13) kJ/mol, and the D phenyl ring: −13(9) kJ/mol, respectively). These interactions seem to be, on average, energetically as favorable as hydrogen bonding with Asp25B (−24(31) kJ/mol), but more stable considering standard deviation of the Ees energy (Supplementary Materials Figure S1). Other contributions worth noting are made by Gly27A hydrogen bonded through CA-H with the ligand’s O21 atom (−15(10) kJ/mol) and Gly27B hydrogen bonded through CA-H with S26 of ligand (−13(10) kJ/mol). Moreover, finally, Ile84A and Arg8A are attracted to (−16(5) kJ/mol) or repulsing from (+13(5) kJ/mol) the C phenyl ring of ligand, respectively, with atom–atom contacts hard to qualify to known categories of intermolecular interactions.

Analogously, for darunavir interacting with the protein, three hydrogen bonds stable over entire simulation were identified (Figure 2 bottom). These are bonds with Asp25A OD2, Asp29B N-H, and Asp30A O. The remaining identified bonds occur less frequently. There is no alternation in hydrogen bonds present, and only one of the possible hydrogen bonding between catalytic residues is observed: OD2-HD2 of Asp25B interacting with only OD2 of Asp25A. Entire darunavir molecule interacts with entire protein with electrostatic energy of −283(52) kJ/mol, and with particular monomers with Ees of −184(44) and −99(35) kJ/mol for monomers A and B, respectively.

Individual residues contributes in somewhat similar fashion to electrostatic interactions with darunavir as it was for JE-2147, especially when Epen is under the focus (Figure 3 and Supplementary Materials Figure S2). However, there are some substantial differences. Slightly different pattern in hydrogen bond formed during the entire simulations has its reflection in electrostatic energies. Only one from the catalytic residues, Asp25A, have strong attractive interaction with darunavir and energy of that interaction is more negative (−92(37) kJ/mol) when comparing with JE-2147 in the 1KZK structure. Interactions with Ile50A and Ile50B are less strong (−29(16) and −36(8) kJ/mol, respectively). Instead, second the most strongly interacting residue is Asp30A (−71(17) kJ/mol), but Asp30B is repulsing (16(7) kJ/mol). Residues Asp29A and Asp29B interact with darunavir similarly as with JE-2147 (−22(4) and −10(14) kJ/mol, respectively). What is completely different is how residues Arg8A, Lys45B, and Arg87B, as well as some other charged residues, interact with darunavir, when compared to analogous residues from the opposite monomer, and to analogous residues from both monomers in protease interacting with JE-2147. These residues have energy of interaction of opposite sign, meaning that, for example, when Arg8B repulse from darunavir (10(2) kJ/mol), residue Arg8A is attractive to it (−12(8) kJ/mol). These are all positively charged residues, not forming any strong atom-atom interactions of hydrogen bond type or other types. At first glimpse the alternation of energy sign is unexpected because darunavir has neutral charge. However, darunavir has twice large dipole moment comparing to JE-2147 due to aminophenyl group (ring A, Figure 1) on one end of the molecule and hydroxyhexahydrofurofuranyl group (rings B and C, Figure 1) on the other end. Residues Arg8B, Lys45A, and Arg86A surround the aminophenyl group and residues Arg8A, Lys45B, and Arg86B located around the other end of darunavir.

### 3.2. Monomer–Monomer Interactions in HIV-1 Protease

**Figure 4:**
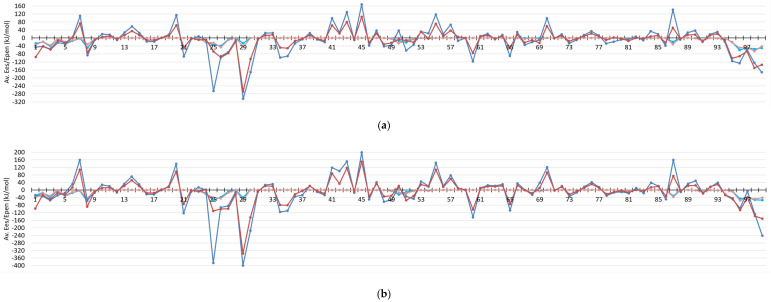
Averaged electrostatic interaction energies (Ees) and penetration contributions to them (Epen) for interactions of a residue from one monomer with all residues of the other monomer for 1KZK (**a**) and 4DQB (**b**) structures. Ees: dark blue—monomer A; dark red—monomer B. Epen: light blue—monomer A; light red—monomer B.

The strength of ligand–protein interactions can be further contrasted with the strength of interactions between monomer A and monomer B of protein. Again, the analysis is based on averaged values of energies computed for 100 snapshots from MD simulations. It appears that the strongest electrostatic attractive or repulsive interactions between a residue from one monomer and all residues of the other monomer are of similar magnitude as interactions of a ligand with a monomer, Figure 4. Moreover, it is remarkable how interaction profiles are similar for 1KZK and 4DQB structures, despite the fact that the MD simulations started from different crystallographic structures, structures contain different ligands, and sequences of proteins are not exactly the same. The proteins come from different subtypes of HIV-1 virus (group M), from subtype B or D for 1KZK and 4DQB structures, respectively, which differ as follows: Leu33→Ile, Pro63→Ile, Val64→Ile, Cys67→Ala, and Cys95→Ala.

The strongest attractive interactions with entire second monomer are observed for residue Asp25 from monomer A, and for Asp29, Asp30, and Phe99 residues from both monomers. These are residues which contribute to the constitution of the active site of the protease (Asp25, Asp29, and Asp30), including one of the catalytic residues (Asp25 A), or belong to the most interpenetrating fragment of a dimer interface, where two β-strands from one monomer and two β-strands from the other one form antiparallel terminal β-sheet.

**Figure 5:**
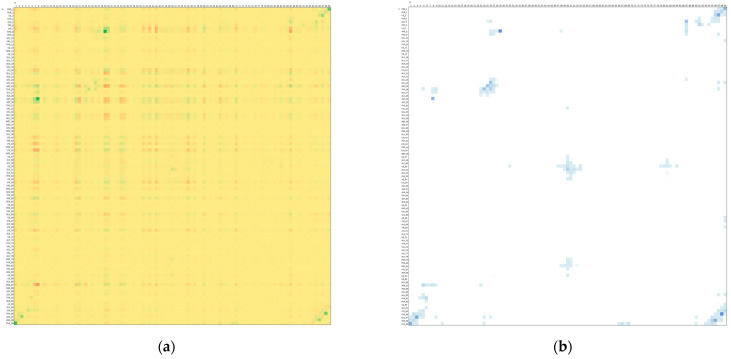
Heat plot for averaged electrostatic interaction energies (**a**) and penetration contributions to it (**b**) for the KZK structure. Scale for (**a**): from green to red, dark green—the most negative values, dark red—the most positive values, and yellow—close to zero values. Scale for (**b**): from blue to white, dark blue—the most positive values, and white—zero values.

Closer look into electrostatic interactions between particular single residues (not a residue and entire monomer) reveals that Asp29 residues forms the strongest residue–residue interactions (Figure 5a). Asp29 from monomer B interacts with Arg8 from monomer A with the most negative Ees, −186(30) kJ/mol for 1KZK and −220(23) kJ/mol for 4DQB, and the pair is followed by its symmetrical counterpart, Asp29A…Arg8B, Ees = −160(70) and −202(34) kJ/mol. Residues Arg8 (B and A) are also the ones which exhibit the largest repulsive interactions with a residue from the other monomer, they interact with Arg87 (A and B) with Ees equal to 64(1) and 55(4) kJ/mol, and to 65(3) and 64(5) kJ/mol for 1KZK and 4DQB, respectively. Interestingly, Asp25 from monomer A (deprotonated) is not interacting particularly strongly with one individual residue, but rather its interactions with many residues are somewhat similar and sum up to a large total negative value of Ees. Ees energies for Asp25 from monomer B (protonated), on the other hand, do not sum up to such large values, most probably because they are not charged anymore, and their long-range electrostatic interactions are weaker. Similar to Asp25A, Asp30 residues interact similarly strongly with many residues from the second monomer. Phe99 residues, on the other hand, interact particularly strongly with Pro1 residues, with Ees in the range of −116 to −170 kJ/mol. The last strongly interacting pairs of residues are Asp98 interacting with Thr96, with Ees in the range of −64 to −100 kJ/mol.

**Figure 6:**
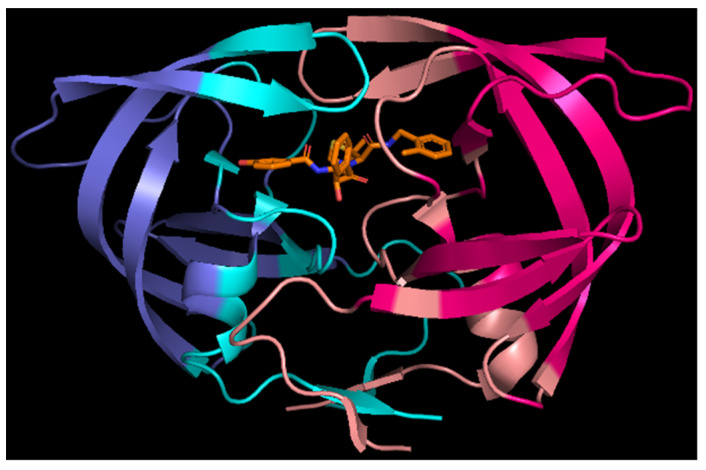
The 1KZK structure with residues exhibiting non-zero penetration energies shown in light blue (monomer A) and light red (monomer B).

All the above-mentioned residues—except for Asp30—besides showing large negative or positive values of Ees, are characterized by significant values of penetration contributions, Epen, to electrostatics interactions. It means their interactions should be considered as short-range, because at least one atom from one residue interpenetrate electron density of the other atom(s) from the second residue. Epen is particularly helpful in identifying pairs of interacting molecular fragments which are interacting with each other directly (Figure 5b and Figure 6, and Supplementary Materials Figures S3 and S4). It is much better than any other simplified method of distance characterization, such as the one shown in Supplementary Materials Figures S3c and S4c. Non-zero Epen, in addition, indicates that other contributions to total interaction energies, such as exchange-repulsion, dispersion, and induction, will be most probably not negligible for that interaction.

Here Epen allows us to easily identify not only regions of active site (residues 23–29) built by mutual interactions of both monomers and regions of N- and C-termini interacting to lock the dimer (residues 1–9 and 90–99), as identified from the Ees analysis above, but also other well-known regions of HIV-1 protease, namely two flaps covering the active site. Residues from 47 to 54 of each monomer directly interact with each other, but their electrostatic interactions are not as strong as in the other mentioned regions. Less strong interactions correspond very well with the loops ability to open spontaneously, what plays a crucial role in the mechanism of substrate binding. Moreover, Epen shows that there are direct interactions between the active site and terminal β-sheet fragments of dimer interface through residues Thr26, Gly27 and Asp29 from one monomer interacting with residues Thr4, Lys7, and Pro9 from the other monomer.

The remaining interacting residues exhibiting larger than zero Epen are the following: Val11, Val32, Ile66, Cys/Ala67, His69, Pro79-Thr80-Pro81-Val82, Ile84, and Arg87. Residues Arg87 was already mentioned in the contexts of strong repulsive interactions with Arg8 from the terminal β-sheet dimer interface. The rest do not interact strongly electrostatically, but, nevertheless, they complete constitution of the dimer interface.

Interestingly, there are many residues with zero charge penetrations, meaning interacting true long-range electrostatic interactions only, showing quite large values of positive or negative energy, ca. +/−80 kJ/mol. Residues acting towards monomer–monomer repulsion are all positively charged: Lys7, Arg14, Lys20, Lys41, Lys43, Lys45, Lys55, Arg57, and Lys70. Residues strengthening monomer–monomer interactions through long-range electrostatic attractions are those negatively charged: Glu21, Glu34, Glu35, Asp60, and Glu65.

All of the above analyses were done on the basis of energies averaged for 100 snapshots from MD. We believe they give more accurate view on interaction energies than analysis of a single structure, let it be from experiment or theoretical modeling. Such analyses are not biased by single realizations of one state from many equal possibilities, what we showed for the 1KZK structure. They also show that that there are residues which exhibit large fluctuations in Ees, but small in overall atom–atom distances: Asp25, Asp29, Ile50, and Arg87, and slightly less visible Thr26 and Asp30. Here rotation of single functional group must influence a lot electrostatic interactions, such as –COO- from Asp29 interacting with guanidine group from Arg8, for which co-planar orientation is the most optimal, or –COOH group from Asp25B forming O-H…O type of hydrogen bond with Asp25A instead with the ligand.

### 3.3. Mutants Disrupting Monomer–Monomer Interactions in HIV-1 Protease

Among many mutants observed in HIV-1 protease, there three often mentioned in the context of weakening of monomer–monomer interactions: Thr26→Ala, Asp29→Asn and Arg87→Lys. Interestingly, all of these mutants are among the residues which exhibit large Ees fluctuations (Supplementary Materials Figure S3b) but relatively small movements of most of the atoms (Supplementary Materials Figure S3d). The mutation of the remaining Asp30 residue is also observed but mention (Asp30→Asn) in the context of ligand binding. Again, Asp30 is experiencing one of the largest fluctuations of Ees, but this time for protein–ligand interactions. Asp29 and Arg87 are also among the ones most strongly interacting electrostatically.

### 3.4. Information Gained When Compared to Conventional Atomic Point-Charge Model of Electrostatics

All of the above analyses were done on the basis of electrostatic interaction energies computed from the UBDB model. To understand if the same information could be gained from much simpler approach, i.e., from the atomic point-charge (PC) model, we computed electrostatic interaction energies using solely point charges, the same which were employed during the MD simulations. Comparison of the interaction profiles for Ees from both methods (Figure 3 and Figure 4, and Supplementary Materials Figures S5 and S6) is giving the clear answer. Ees energies from PC are much larger in magnitude (almost two times) but are observed almost only for charged residues. All the fine details about electrostatic interactions involving neutral residues are lost, and information on which residues interact with each other via direct contact and which through truly long-range is not accessible directly from electrostatics analysis. Electrostatic energies from the UBDB model are much better balanced, and richer in information.

## 4. Conclusions and Outlook

Electrostatic interactions between protein monomer–monomer and protein–ligand were analyzed by using aspherical atom databank (UBDB) over snapshots from trajectory obtained from molecular dynamic simulation. The electrostatic interaction is a non-negligible component of the interaction energy driving the strength of HIV-1 protease inhibition and must be embraced when designing new inhibitors. The model applied in our work takes into account the asphericity of atoms by using aspherical electron density fragments specific for particular atom types stored in the UBDB. Unlike point-charge models, the UBDB approach takes into account the directionality of the atom–atom interactions and charge penetration. The model is applicable to microscopic analysis of structure–function activity in biological molecules. Moreover, the electrostatic interactions are much stronger than vdW interactions and simultaneously can provide sufficient description of the binding strength, including the consequences of the fact that molecules have their volume (penetration contribution). Thus, electrostatics itself can be used as a sole scoring function. The UBDB model may also serve as a base for building more universal scoring functions, as it is compatible with any other physically meaningful energy term developed following quantum-mechanical schemes of interaction energy decomposition, such as dispersion [55] or polarization [56]. Expansion of protein–ligand interaction analyses by analysis of monomer–monomer interactions in multimeric proteins gives wider contexts for protein–ligand interactions. Moreover, the inclusion of dynamic aspects of protein–ligand complex helps to understand where the hot spots that are important for dimerization and possible mutations are. Therefore, the new designed molecules will be not only effective against wild-type protease but also against its drug-resistant mutant variants.

In the next step of the research work, more emphasis on the role of electrostatic interactions in various kinds of protease mutants is necessary, including both, those related with dimer destabilization, and those influencing ligand binding. This, along with deeper studies of the protein dynamic in its apo- and ligand-bound forms, in our opinion, will bring a much deeper understanding of the drug-resistance mechanisms.

## Data Availability

The raw data supporting reported results can be obtained from the authors upon request.

## Supplementary Materials

The following are available online. Figure S1: 1D profiles for averaged standard deviations of electrostatic interaction energies (a), averaged atom-atom distances summed per residue-ligand pair (b), averaged standard deviations of atom-atom distances summed per residue-ligand pair (c), and penetration contributions to electrostatic energies (d) for the 1KZK structure; Figure S2: 1D profiles for standard deviations of electrostatic interaction energies (a), averaged atom-atom distances summed per residue-ligand pair (b), averaged standard deviations of atom-atom distances summed per residue-ligand pair (c), and penetration contributions to electrostatic energies (d) for the 4DBQ structure; Figure S3: Heat plots and 1D profiles for averaged electrostatic interaction energies (kJ/mol) (a), averaged standard deviations of electrostatic interaction energies (kJ/mol) (b), averaged atom-atom distances summed per residue-reside pair (Å) (c), standard deviations of atom-atom distances summed per residue-residue pair (Å) (d), and penetration contributions to electrostatic energies (kJ/mol) (e) for the 1KZK structure; Figure S4: 1D profiles for averaged electrostatic interaction energies (kJ/mol) (a), standard deviations of electrostatic interaction energies (kJ/mol) (b), averaged atom-atom distances summed per residue-reside pair (Å) (c), standard deviations of atom-atom distances summed per residue-residue pair (Å) (d), and penetration contributions to electrostatic energies (kJ/mol) (e) for the 4DBQ structure; Figure S5: Averaged electrostatic interaction energies (Ees, kJ/mol) computed from the PC model for interactions of a ligand with each residue of a monomer for the 1KZK (a) and 4DQB (b) structures. Dark green: monomer A, dark orange: monomer B. Averaged electrostatic interaction energies (Ees) computed from the PC model for interactions of a residue from one monomer with all residues of the other monomer for the 1KZK (c) and 4DQB (d) structures.; Figure S6: 1D profiles for averaged standard deviations of electrostatic interaction energies (kJ/mol) computed from the PC model for the 1KZK (a), (c) and 4DBQ (b), (d) structures.
