# Supplementary material for: Combining Molecular Dynamic Information and an Aspherical-Atom Data Bank in the Evaluation of the Electrostatic Interaction Energy in Multimeric Protein-Ligand Complex: A Case Study for HIV-1 Protease
Source: Molecules. 2021 Jun 24;26(13):3872. doi: 10.3390/molecules26133872 (PMC8270314; doi:10.3390/molecules26133872)
Supplement: Supplementary file 1 [file molecules-26-03872-s001.zip › molecules-1237461-supplementary.pdf]

Supporting Information

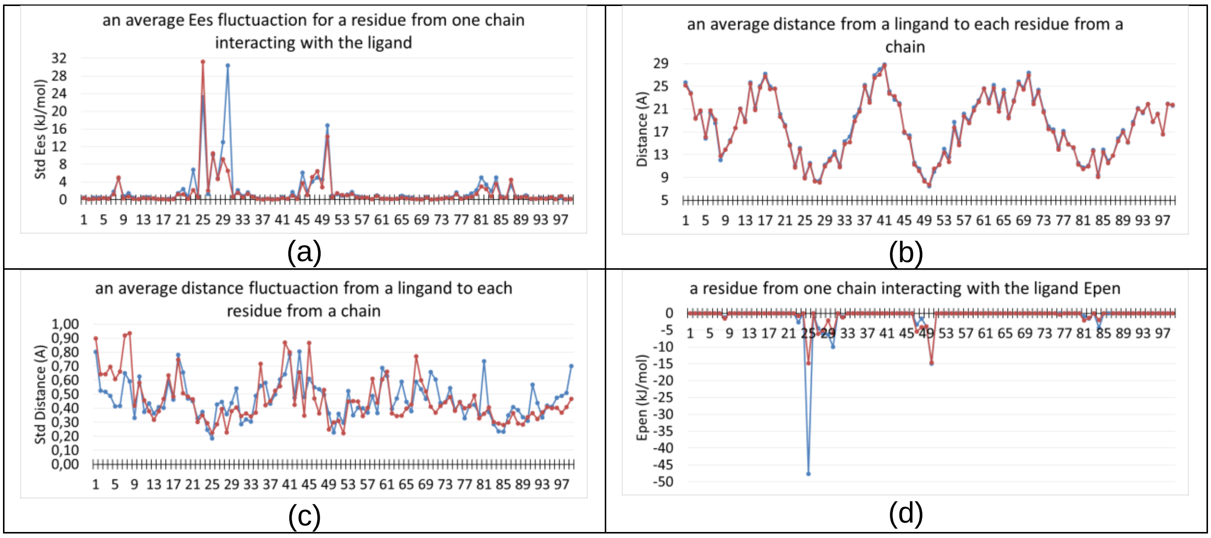

Figure S1: 1D profiles for averaged standard deviations of electrostatic interaction energies (a), averaged atom-atom distances summed per residue-ligand pair (b), averaged standard deviations of atom-atom distances summed per residue-ligand pair (c), and penetration contributions to electrostatic energies (d) for the 1KZK structure. Blue: monomer A, red: monomer B.

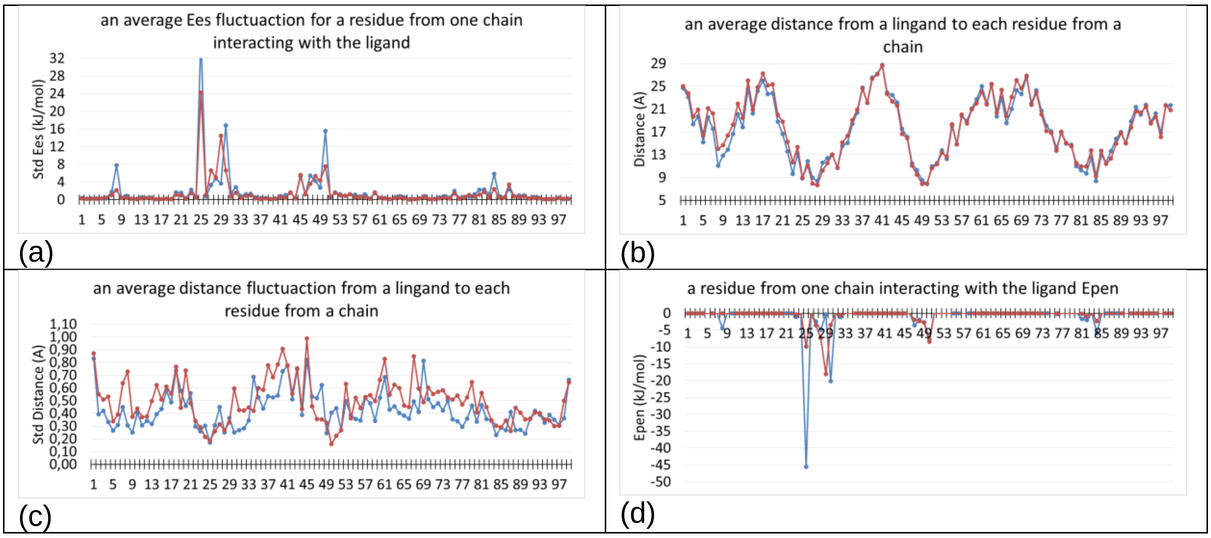

Figure S2: 1D profiles for standard deviations of electrostatic interaction energies (a), averaged atom-atom distances summed per residue-ligand pair (b), averaged standard deviations of atom-atom distances summed per residue-ligand pair (c), and penetration contributions to electrostatic energies (d) for the 4DBQ structure. Blue: monomer A, red: monomer B.

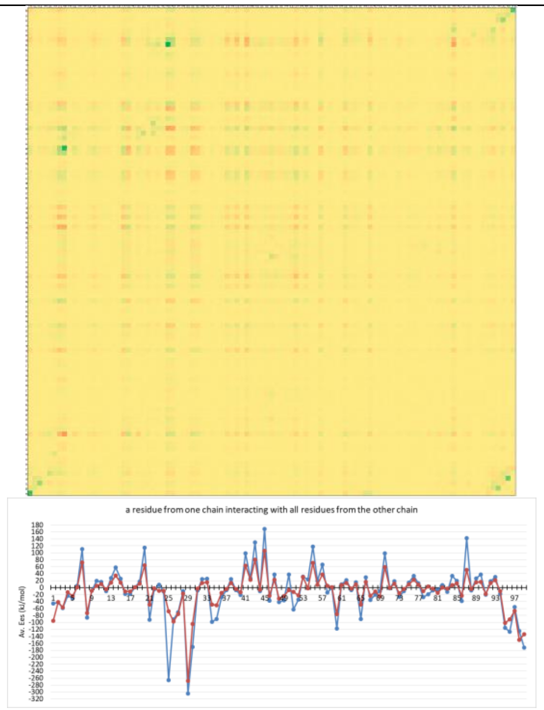
a residue from one chain interacting with all residues from the other chain
Av. Ees (kJ/mol)

(a)

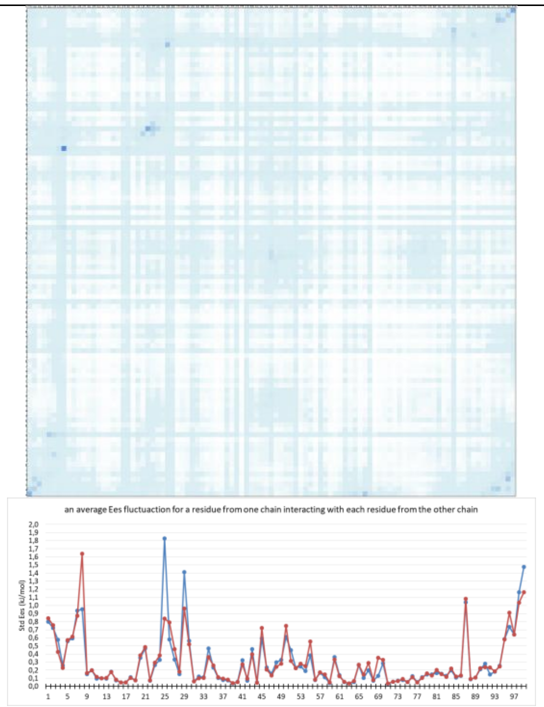
an average Ees fluctuaction for a residue from one chain interacting with each residue from the other chain
Std Ees (kJ/mol)

(b)

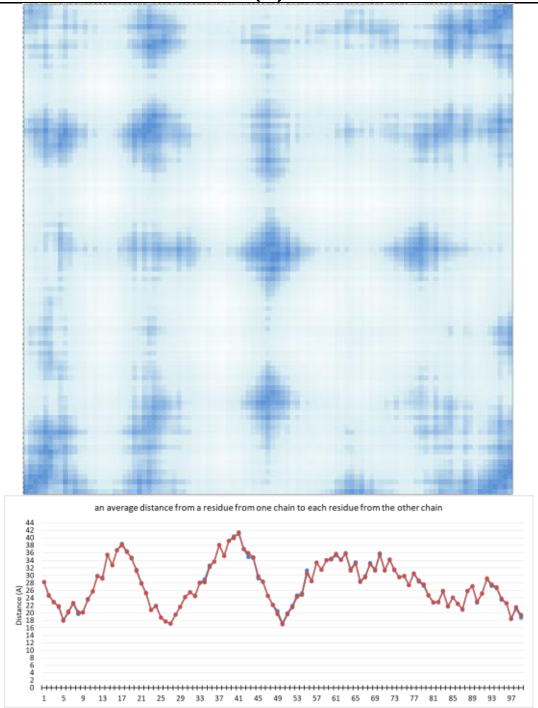
an average distance from a residue from one chain to each residue from the other chain
Distance (A)

(c)

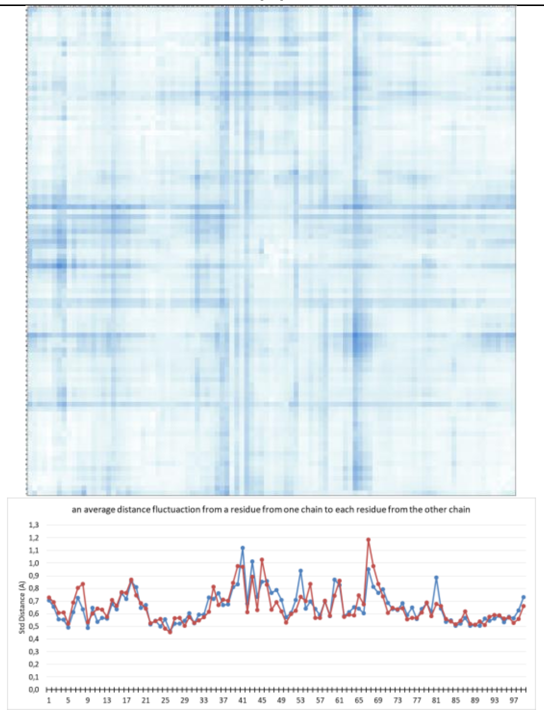
an average distance fluctuaction from a residue from one chain to each residue from the other chain
Std Distance (A)

(d)

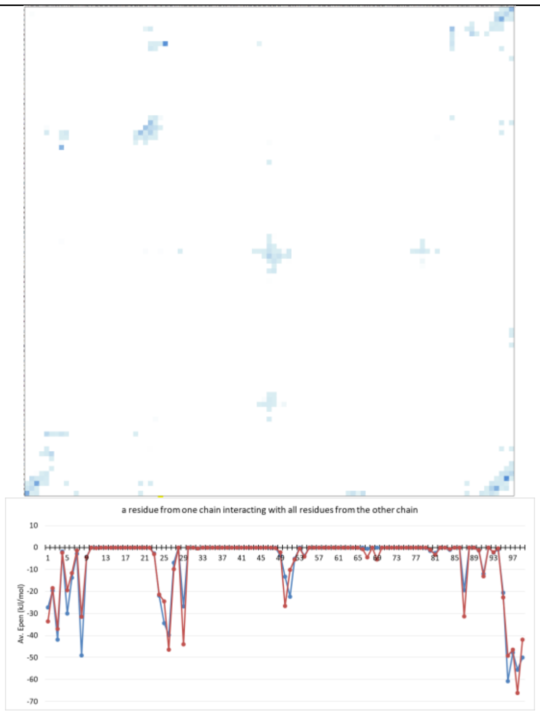

(e)

Figure S3: Heat plots and 1D profiles for averaged electrostatic interaction energies (kJ/mol) (a), averaged standard deviations of electrostatic interaction energies (kJ/mol) (b), averaged atom-atom distances summed per residue-reside pair (Å) (c), standard deviations of atom-atom distances summed per residue-residue pair (Å) (d), and penetration contributions to electrostatic energies (kJ/mol) (e) for the 1KZK structure. For detailed descriptions see Figures 4 and 5.

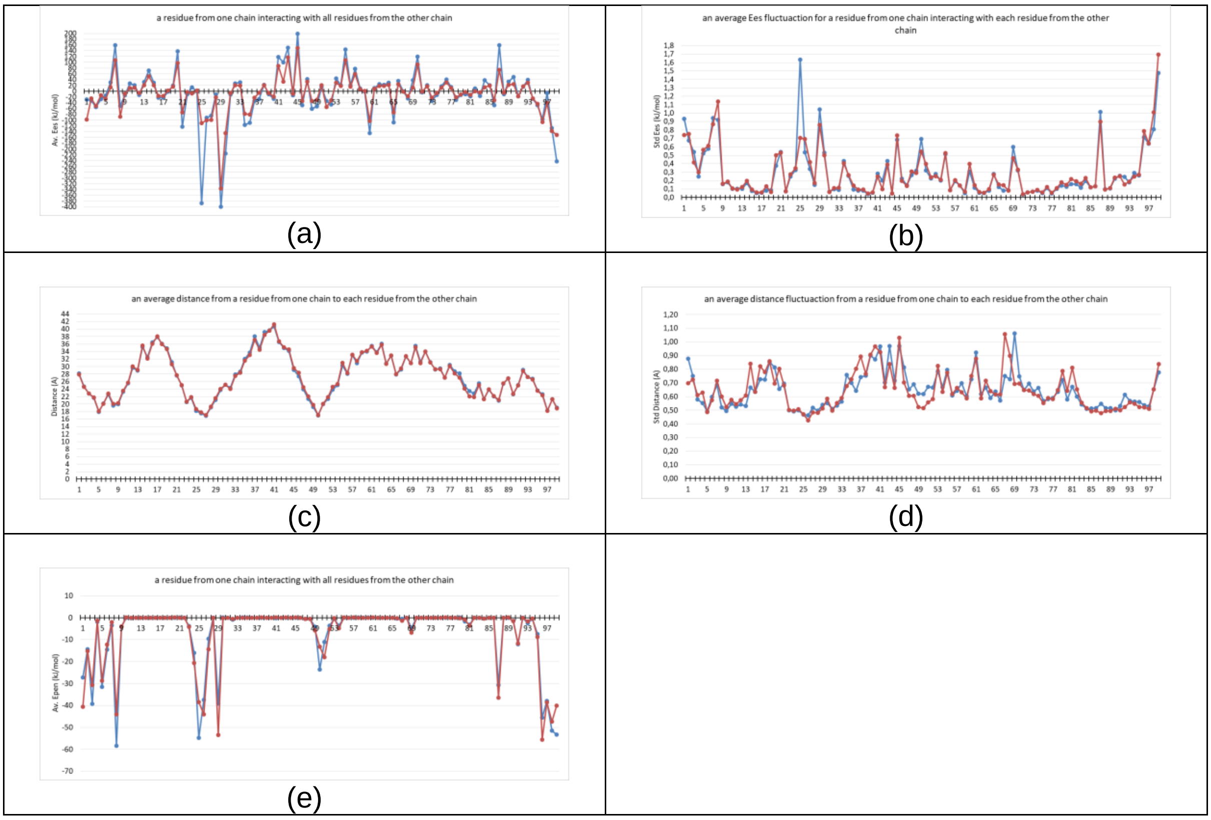

Figure S4: 1D profiles for averaged electrostatic interaction energies (kJ/mol) (a), standard deviations of electrostatic interaction energies (kJ/mol) (b), averaged atom-atom distances summed per residue-reside pair (Å) (c), standard deviations of atom-atom distances summed per residue-residue pair (Å) (d), and penetration contributions to electrostatic energies (kJ/mol) (e) for the 4DBQ structure. For detailed descriptions see Figures 4 and 5.

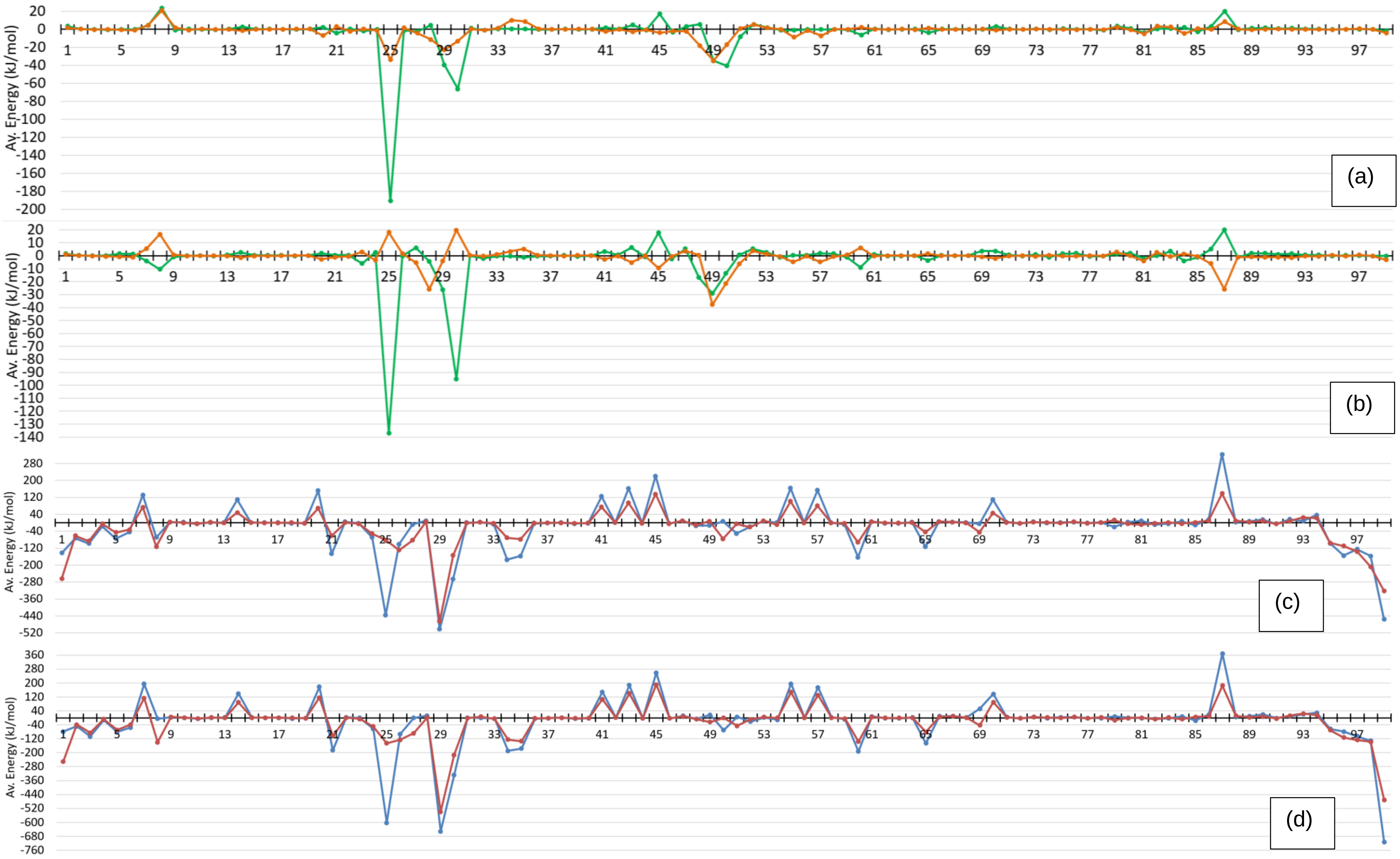
Av. Energy (kJ/mol)
(a)
Av. Energy (kJ/mol)
(b)
Av. Energy (kJ/mol)
(c)
Av. Energy (kJ/mol)
(d)

Figure S5: Averaged electrostatic interaction energies (Ees, kJ/mol) computed from the PC model for interactions of a ligand with each residue of a monomer for the 1KZK (a) and 4DQB (b) structures. Dark green: monomer A, dark orange: monomer B. Averaged electrostatic interaction energies (Ees) computed from the PC model for interactions of a residue from one monomer with all residues of the other monomer for the 1KZK (c) and 4DQB (d) structures. Dark blue: monomer A, dark red: monomer B.

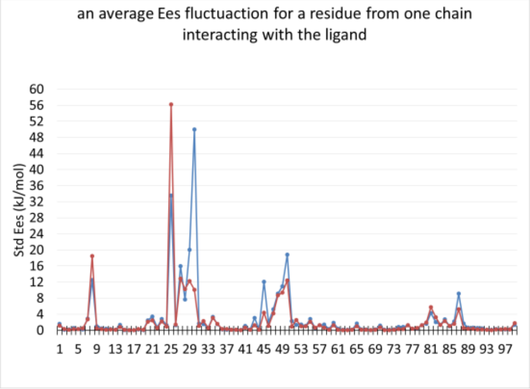

(a)

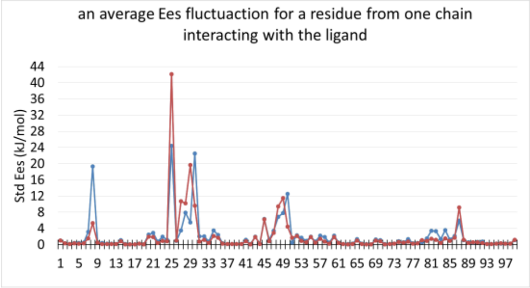

(b)

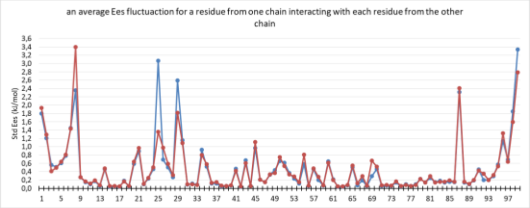

(c)

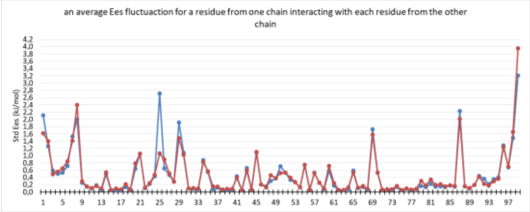

(d)

Figure S6: 1D profiles for averaged standard deviations of electrostatic interaction energies (kJ/mol) computed from the PC model for the 1KZK (a), (c) and 4DBQ (b), (d) structures. Blue: monomer A, red: monomer B.

.
